# African regional progress and status of the programme to eliminate lymphatic filariasis: 2000–2020

**DOI:** 10.1093/inthealth/ihaa058

**Published:** 2020-12-22

**Authors:** Kebede Deribe, Didier K Bakajika, Honorat Marie-Gustave Zoure, John O Gyapong, David H Molyneux, Maria P Rebollo

**Affiliations:** Expanded Special Project for Elimination of NTDs, World Health Organization Regional Office for Africa, Cité du Djoué, P.O. Box 06 Brazzaville, Republic of Congo; Brighton and Sussex Centre for Global Health Research, Department of Global Health and Infection, Brighton and Sussex Medical School, Brighton, BN1 9PX, UK; Expanded Special Project for Elimination of NTDs, World Health Organization Regional Office for Africa, Cité du Djoué, P.O. Box 06 Brazzaville, Republic of Congo; Expanded Special Project for Elimination of NTDs, World Health Organization Regional Office for Africa, Cité du Djoué, P.O. Box 06 Brazzaville, Republic of Congo; University of Health and Allied Sciences, PMB 31, Ho, Volta Region, Ghana; Liverpool School of Tropical Medicine, Pembroke Pl, Liverpool L3 5QA, UK; Expanded Special Project for Elimination of NTDs, World Health Organization Regional Office for Africa, Cité du Djoué, P.O. Box 06 Brazzaville, Republic of Congo

**Keywords:** Africa, elimination, LF, lymphatic filariasis, mass drug administration, neglected tropical diseases, NTDs

## Abstract

To eliminate lymphatic filariasis (LF) by 2020, the World Health Organization (WHO) has launched a campaign against the disease. Since the launch in 2000, significant progress has been made to achieve this ambitious goal. In this article we review the progress and status of the LF programme in Africa through the WHO neglected tropical diseases preventive chemotherapy databank, the Expanded Special Project for Elimination of Neglected Tropical Diseases (ESPEN) portal and other publications. In the African Region there are 35 countries endemic for LF. The Gambia was reclassified as not requiring preventive chemotherapy in 2015, while Togo and Malawi eliminated LF as a public health problem in 2017 and 2020, respectively. Cameroon discontinued mass drug administration (MDA) and transitioned to post-MDA surveillance to validate elimination. The trajectory of coverage continues to accelerate; treatment coverage increased from 0.1% in 2000 to 62.1% in 2018. Geographical coverage has also significantly increased, from 62.7% in 2015 to 78.5% in 2018. In 2019, 23 of 31 countries requiring MDA achieved 100% geographic coverage. Although much remains to be done, morbidity management and disability prevention services have steadily increased in recent years. Vector control interventions conducted by other programmes, particularly malaria vector control, have had a profound effect in stopping transmission in some endemic countries in the region. In conclusion, significant progress has been made in the LF programme in the region while we identify the key remaining challenges in achieving an Africa free of LF.

## Introduction

In the past 20 y, momentum to eliminate lymphatic filariasis (LF) in Africa has significantly improved as a result of development of single-dose treatment strategies, point-of-care diagnostic tools, generous donations of medicines from pharmaceutical companies and financial support for programme implementation from the donor community.^1^ The African Region (AFRO) of the World Health Organization (WHO) includes 38.3% of the global population and 31 of the 49 countries requiring preventive chemotherapy for LF, a debilitating vector-borne infection that affects the poorest populations.^2^ In Africa it is caused by *Wuchereria bancrofti* and is mainly transmitted to humans by mosquito species belonging to *Anopheles* and *Culex*.^3^ In 2000 there were 39 countries believed to be endemic for LF in the WHO AFRO. By that same year, 405.9 million people in 39 countries in Africa were estimated to require preventive chemotherapy. However, according to progress report of the Global Programme to Eliminate Lymphatic Filariasis for 2000–2009,^4^ the evidence for active transmission of LF in many of the 39 endemic countries was weak and some probably did not require mass drug administration (MDA). The status of five countries (Burundi, Cape Verde, Mauritius, Rwanda and Seychelles) were reviewed in 2011 and were reclassified as non-endemic, reducing the number of endemic countries in Africa to 34 (inclusion of South Sudan following independence in 2011 now makes 35).^5^ Of the 35 LF-endemic countries in the region, 2 have eliminated LF as a public health problem (Malawi and Togo), The Gambia was reclassified as not requiring preventive chemotherapy and Cameroon is under post-MDA surveillance to validate if elimination targets have been achieved. In the remaining 31 countries there remain 341.4 million people who require preventive chemotherapy for LF^2^ (Table 1).

**Table 1.** Lymphatic filariasis implementation status in the WHO AFRO as of January 2020

Indicator	Number of countries
Countries verified as eliminating LF as a public health problem	2: Malawi and Togo^2,6^
Countries stopped MDA (under surveillance)	1: Cameroon^2^
Countries implementing MDA with 100% geographical coverage	23: Benin, Burkina Faso, Chad, Comoros, Congo, Côte d'Ivoire, Eritrea, Ethiopia, Ghana, Guinea, Guinea-Bissau, Kenya, Liberia, Mali, Mozambique, Niger, Senegal, Sao Tome and Principe, Sierra Leone, Uganda, United Republic of Tanzania, Zambia and Zimbabwe^6^
Countries implementing MDA in only part of the geographical area considered in need of treatment	6: Angola, Central African Republic, Democratic Republic of the Congo, Madagascar, Nigeria and South Sudan^6^
Countries reclassified as not requiring preventive chemotherapy	1: The Gambia^7^
Countries where MDA is not yet started	2: Equatorial Guinea and Gabon^2^
Countries with mapping gap	1: Equatorial Guinea^6^

## Progress and achievements

### Mapping the geographical distribution of LF

Mapping the geographical distribution of a disease is a key step prior to the implementation of any public health intervention. In 2000, the first LF mapping was initiated in Africa and by 2001, four countries had already completed mapping (Benin, Burkina Faso, Ghana and Togo).^8^ Mapping was subsequently conducted in the remaining countries within the region from 2002 to 2012 with the support of different stakeholders and from 2013 to 2015 under the leadership of the WHO AFRO Mapping Project. The AFRO Mapping Project accelerated the mapping of LF in many countries. Nevertheless, mapping was delayed in three countries (Central African Republic, Mauritania and South Sudan) due to security-related challenges. It was only in 2018 and 2019 that these countries were able to complete LF mapping under the leadership of the WHO Expanded Special Project for the Elimination of Neglected Tropical Diseases (ESPEN). Currently only one implementation unit (Annanbon) in Equatorial Guinea is unmapped because of its inaccessibility.

### MDA

**Figure 1.**
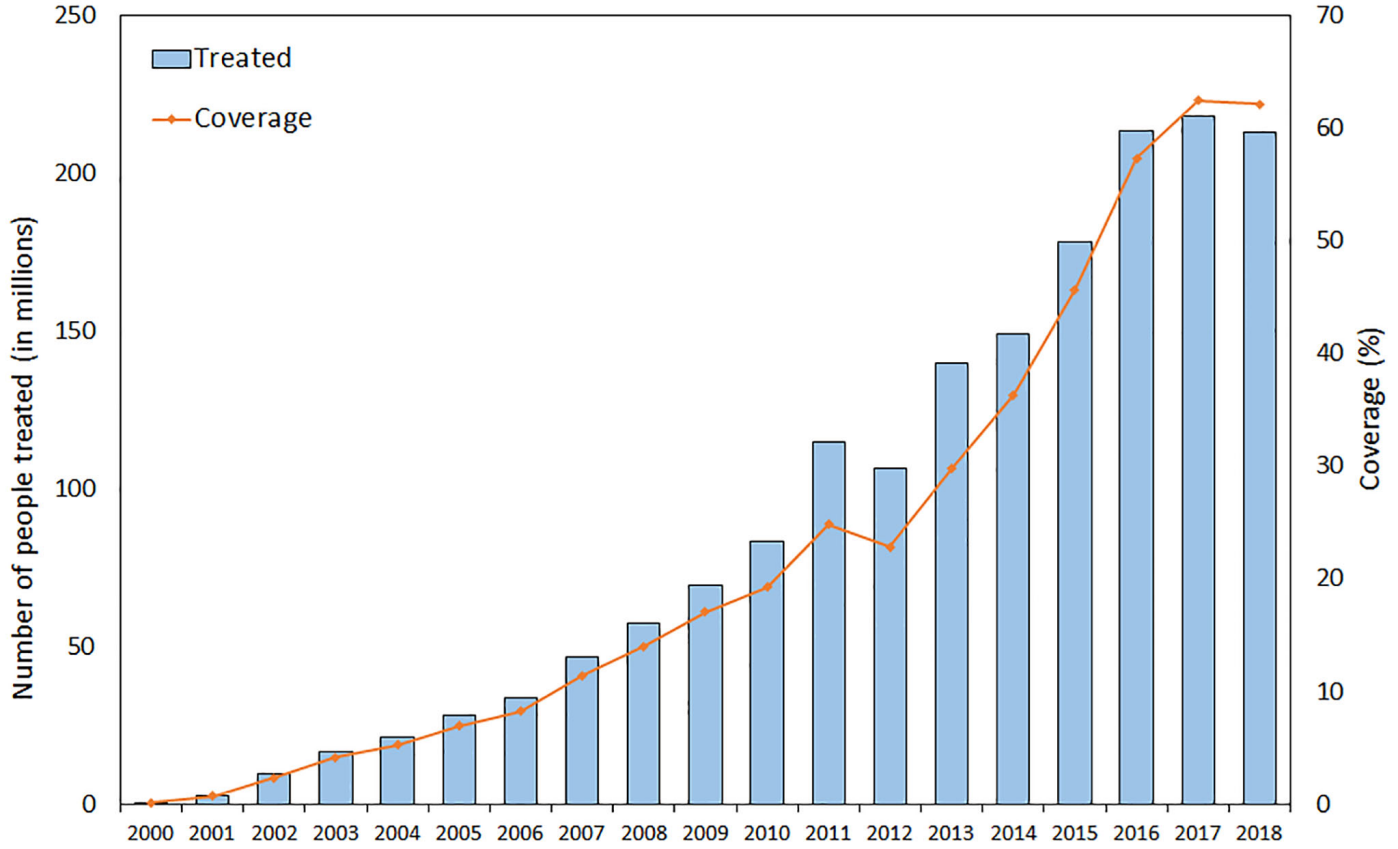
Number of people treated and progress in preventive chemotherapy coverage, 2000–2018. Data for this figure were accessed from the neglected tropical diseases PCT databank.^9^

MDA with albendazole in combination with either ivermectin or diethylcarbamazine (the latter combination in countries non-co-endemic for onchocerciasis) or albendazole alone were implemented progressively in endemic counties. In 2000, the AFRO treated only 363 607 people compared with 212.7 million in 2018, according to data from the WHO neglected tropical diseases preventive chemotherapy (PCT) databank^9^ (Figure 1).^10,11^ By the beginning of 2020, a total of 23 of 31 countries had implemented at least one round of MDA in all endemic implementation units (IUs).^11^ Only two countries (Equatorial Guinea and Gabon) have yet to commence MDA.^11^ The trajectory of coverage continues to increase, from 0.1% in 2000 to 62.1% in 2018. Similarly, the proportion of countries that have achieved national effective coverage (defined as coverage of at least 65% for LF) has increased from 68.1% in 2015 to 90.2% in 2018.^7,11^ The proportion of IUs delivering preventive chemotherapy in IUs requiring MDA reached 78.5% in 2018, up from 62.7% in 2015.^7,11^ The LF programme is one the biggest deworming programmes in Africa. Since all LF-endemic countries are also endemic for soil-transmitted helminthiasis (STH), many school-age children have benefited through the LF programme, although the impact has yet to be quantified.^12^

### Triple therapy

The treatment, known as triple therapy, involves a combination of ivermectin, diethylcarbamazine citrate and albendazole (IDA) in areas where onchocerciasis is not endemic.^13^ Studies have demonstrated that IDA is superior to the previous standard regimens of diethylcarbamazine plus albendazole (two-drug regimen),^14,15^ as IDA clears microfilaria more efficiently from the blood than the two-drug regimen and is equally safe.^14,16^ In 2017 the WHO released a new guideline recommending the IDA regimen as an alternative treatment strategy in certain settings where onchocerciasis is not endemic.^13^ In May 2018 in Nairobi, the WHO convened a technical meeting on IDA in Africa to review the progress of seven countries eligible for IDA for the elimination of LF and to guide the implementation of the strategy. Kenya was the first country in the region to implement the strategy in three subcounties targeting 278 291 individuals.^2^ A year later, São Tomé and Príncipe treated 148 460 of 206 194 individuals with the triple-drug regimen, reaching national coverage of 72%. All the treated IUs achieved effective coverage. Building on that success, Comoros, Eritrea and Madagascar have planned the implementation of IDA in 2020. With more than four rounds of MDA with the double therapy, Zambia was not found to be eligible for IDA, while Zimbabwe, with two rounds of MDA, will re-evaluate its situation in 2020 before deciding if IDA should be implemented.

### Transmission assessment survey (TAS)

A TAS is recommended in an evaluation unit (EU) after a successful preliminary survey (pre-TAS) to determine when infections have been reduced below target thresholds (interruption of transmission) and MDA can stop after at least five consecutive rounds with effective coverage. It is recommended to conduct three TASs with an interval of 2 y between each. As of December 2019, a TAS had been conducted in 370 EUs covering 1533 IUs in 16 countries. In total, TAS1 has been implemented in 749 IUs, TAS2 in 581 IUs and TAS3 in 203 IUs representing 31.6%, 24.5% and 8.6%, respectively, of the 2372 endemic IUs, with the technical and financial support of either the WHO or other partners.

### Morbidity management and disability prevention

Morbidity alleviation, the second pillar of the global program, was almost non-existent in 2000. By 2015, 11 endemic countries were reporting hydrocele and lymphedema patients, while 12 countries reported on morbidity management and disability prevention (MMDP) services. There has been steady progress over the years, with 22 and 23 countries reporting on lymphedema and hydrocele cases, respectively, as of the beginning of 2019. A study in Malawi documented that hydrocele surgery improves quality of life significantly at 6 months post-surgery.^17^ Another study showed that the lifetime benefits of hydrocelectomy by far exceed the costs of repairing hydroceles.^18^ MMDP activities have generally lagged behind MDA and there is a need to improve the coverage of MMDP services and the number of countries implementing these patient-oriented morbidity interventions.

### Vector control

The WHO Position Statement on Integrated Vector Management (IVM) recommends integrated vector control of malaria and LF.^19^ These recommendations are pertinent in Africa because *Anopheles* species are the common vectors of both infections and vector control interventions, particularly insecticide-treated mosquito nets (ITNs) and indoor residual spraying, impact the transmission of both *Plasmodium* and *Wuchereria*, shown initially in the Solomon Islands.^20,21^ Studies have documented that the prevalence of *W. bancrofti* infection in The Gambia was among the highest in Africa in the 1950s.^22,23^ Nonetheless, different surveys conducted in 1975 and 1976 revealed a significant decline in endemicity in the absence of MDA.^24^ A study conducted in 2013, using the TAS methodology, confirmed the transmission interruption of *W. bancrofti* in The Gambia.^25^ The studies attributed the decline in prevalence to a significant reduction in mosquito density through the widespread use of ITNs as part of the national malaria control programme, which is in accordance with the results of a study conducted in Zambia.^26^

Another study highlighted the role of competitive exclusion in the low endemicity of LF in Central Africa.^27^ In ecology, competitive exclusion states that two species competing for the same resources cannot stably coexist when all other ecological factors are constant. When one species has even the slightest advantage over the other, then one will dominate in the long term or one of the competitors will adapt via a behavioural shift towards a different ecological niche.^27^ Six filarial parasites can infect people in sub-Saharan Africa: *W. bancrofti, Onchocerca volvulus, Loa loa, Mansonella perstans, Mansonella streptocerca* and *Dracunculus medinensis*. Although there is some degree of co-endemicity among these filarial parasites, there are also areas where competitive exclusion is proposed to reduce this co-endemicity, which reduces the likelihood of competition for resources given the distribution of adult and microfilaria larvae into separate niches in the human host through spatial and temporal segregation, as shown by the different niches of adult filariae and the different periodicities of the microfilariae or sites (peripheral blood or skin) while the vectors of African filariae are from different insect groups (mosquitoes, *Chrysops, Simulium* and *Culicoides*) with different biting habits.^25^

### Elimination of LF as a public health problem

**Figure 2.**
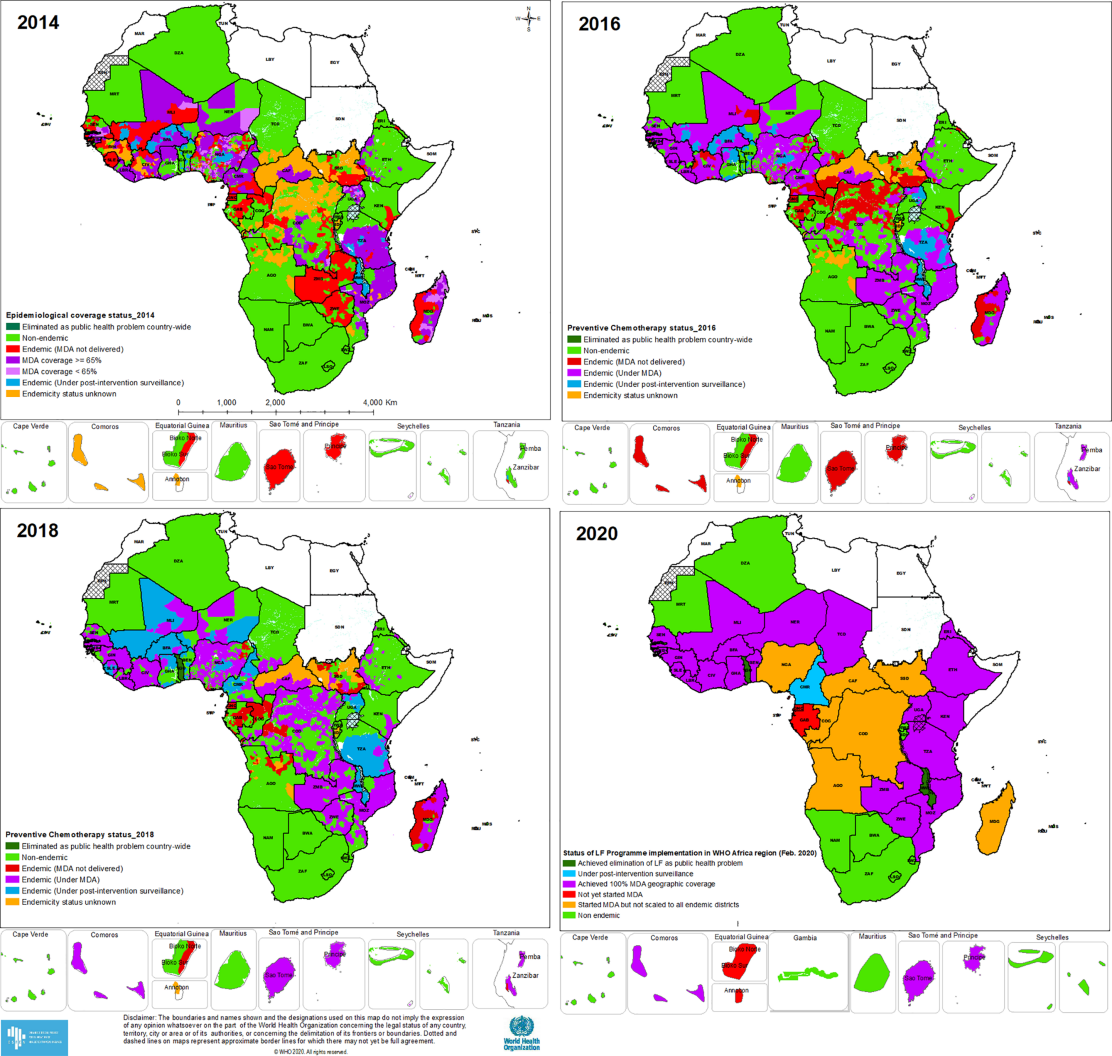
Status of LF programme implementation in Africa. Data for developing the maps were accessed from the ESPEN portal.^28^

Two countries, Togo and Malawi, from the WHO AFRO eliminated LF as a public health problem in 2017 and 2020, respectively. In 2019, Cameroon discontinued MDA programmes and transitioned to post-elimination surveillance. Several countries (Benin, Burkina Faso, Ethiopia, Ghana, Madagascar, Mali, Nigeria, Senegal, Uganda and the United Republic of Tanzania) have stopped MDA in at least one EU based on data from the ESPEN portal.^28^ (Figure 2)

## Programme challenges

Despite these achievements in the AFRO, several challenges have been encountered, including security problems reducing access and political instability in some countries, that has contributed to delayed mapping of LF and the conducting of TASs. For instance, Central African Republic, Mauritania and South Sudan completed their mapping in 2018 and 2019. Another challenge to be highlighted is the co-endemicity with *L. loa* in most of the countries in Central Africa, which has contributed to delays in the implementation of MDA in some countries until the WHO recommended biannual MDA with albendazole in LF–*Loa* co-endemic settings.^13,27,29^ Implementation of an ivermectin-based community treatment strategy for the elimination of LF has been delayed in Central Africa because of the occurrence of serious adverse events, including post-ivermectin encephalopathy and death, in persons with high levels of circulating *L. loa* microfilariae.^30^^–^^32^*L. loa* cross-reactivity continued to be a challenge for mapping LF in Central Africa. Studies have documented that antigen-based tests such as the Filariasis Test Strip (FTS)^33,34^ provide false-positive results in areas where *L. loa* is highly prevalent, indicating the need for developing a confirmatory mapping strategy for such scenarios.^35^

MDA is designed for rural populations and poses significant challenges when it is implemented in urban areas due to population density, population mobility and challenges on how to define target areas for implementation of the strategy.^36,37^ Re-evaluation of MDA is recommended given the challenge of achieving effective coverage of MDA in such settings.^36^ While ongoing transmission of *W. bancrofti* in cities in East Africa^36^ is possible, in West Africa, transmission might not be ongoing due to the fact that *Culex* sp. mosquitoes are inefficient vectors of *W. bancrofti* in West Africa.^38,39^ Prevalences of <1% were registered in many of the cities, including Monrovia, Freetown, Conakry and urban areas in Kano State in Nigeria.^37,40,41^ Therefore re-evaluation of the current endemicity status of the urban areas in West Africa is important.

Much of the focus of the LF elimination programme has been on MDA and there had been little progress in the implementation of the second pillar, morbidity management and disability prevention. It is only in recent years that countries are focusing emphasis on this necessary intervention and scaling up the services for those in need. The increasing level of resistance to present pyrethroid-based insecticides is another challenge to the vector control aspect of the programme.^42^

## LF elimination prospects for 2030

The new Neglected Tropical Diseases Roadmap targets validation by 2030 of elimination of LF as a public health problem from 81% of the endemic countries globally.^43^ In the AFRO, building on the lessons learned in LF elimination from Malawi and Togo, endemic countries in the region should use the opportunity to defeat LF once and for all. Lessons learned from Togo demonstrated that strong political commitment, integration with existing health interventions, innovative resources mobilization and very strong partnerships were success factors.^44^ The country secured joint malaria/LF funding with grants from the Global Fund to Fight AIDS, Tuberculosis and Malaria (GFATM), which facilitated the implementation of a joint programme for the two diseases.^44^

## Conclusions

Over the past 20 y significant progress has been achieved in the AFRO of the WHO as a result of innovation in treatment and diagnostics, the provision of donated medicines and financial support and strong partnerships allied with greater country commitments. Several key milestones have been achieved: mapping has almost been completed; MDA has been scaled up in almost all countries, with the majority of the countries reaching 100% geographical coverage, to accelerate elimination and IDA MDA has been started in the region. Most importantly, two countries have eliminated LF as a public health problem in the region. Despite this significant progress, there are remaining challenges that need to be addressed to see Africa free of LF.
